# Analytical solution for the motion of a pendulum with rolling wheel: stability analysis

**DOI:** 10.1038/s41598-022-15121-w

**Published:** 2022-07-24

**Authors:** Galal M. Moatimid, T. S. Amer

**Affiliations:** 1 Department of Mathematics, Faculty of Education, Ain Shams University, Cairo, Egypt; 2 Department of Mathematics, Faculty of Science, Tanta University, Tanta, 31527 Egypt

**Keywords:** Mathematics and computing, Physics

## Abstract

The current work focuses on the motion of a simple pendulum connected to a wheel and a lightweight spring. The fundamental equation of motion is transformed into a complicated nonlinear ordinary differential equation under restricted surroundings. To achieve the approximate regular solution, the combination of the Homotopy perturbation method (HPM) and Laplace transforms is adopted in combination with the nonlinear expanded frequency. In order to verify the achievable solution, the technique of Runge–Kutta of fourth-order (RK4) is employed. The existence of the obtained solutions over the time, as well as their related phase plane plots, are graphed to display the influence of the parameters on the motion behavior. Additionally, the linearized stability analysis is validated to understand the stability in the neighborhood of the fixed points. The phase portraits near the equilibrium points are sketched.

## Introduction

From a perspective practical interpretation, numerous manufactured structures, like clocks, percussive instruments, rollers, coasters, and earthquakes gravitational anomalies, incorporate pendulums. Additionally, engineers attempt to understand the physics of pendulums, gravity, inertia, and centripetal force. A pendulum is employed to help the tempo of music. One of the most studied motions in practical physics and engineering is the oscillatory motion of a simple pendulum. Motion plays a significant part in the history of physics as well as in the general themes in textbooks and mechanics programs for undergraduates. Therefore, the simple pendulum is the most standard example in mechanics and its study relaxes the beginnings of classical mechanics. In several textbooks and engineering problems, the periodic motion is established by smaller angle fluctuations when using a basic pendulum^1^. Outside this restriction, its governing equation is a nonlinear one. Nevertheless, there is an exact analytical solution for this problem in an integral formula^2^. Therefore, obtaining an accurate bounded approximate solution is actually cooperative^3,4^. A simple pendulum is the most fundamental, inclusive system and serves as the foundation for many complicated applications. Its importance in understanding nonlinear occurrences about us is recognized in engineering disciplines as well as elementary areas such as physics and chemistry. Interestingly, a lower dimensional compound system, such as a double pendulum, swinging Atood’s machine^5^, elastic pendulum^6–8^, and spring-mass-pendulum^9,10^ are sufficient to demonstrate an extensive range of non-trivial phenomena such as continuous processes and diverse categories of resonance. Several machine parts^5–10^ are made up of a simple pendulum and another oscillating system such as a pendulum travelling in a plane or a pendulum revolving with different trajectories of their pivots. In such a mechanical system, the auto parametric resonance is quite essential. The phenomena of a normal mode of adjusting fundamental systems frequencies become unstable. The planar flexible pendulum, two degree of freedom system, is a basic weak low-dimensional sample of an auto parametric organization. Because of the importance of the simple pendulum problem, the present study looked at when it was coupled to a lightweight spring.

The appearance of ordinary and partial differential equations plays a significant part in various fields of sciences, practical physics, chemistry, mathematics, and biology. Qualitatively, there is physical relevance of the situation that determines the dynamical behaviors. Population expansion, potential fields, electric circuits, tree biological nature, and so on are all examples of the uses of practical physics. Differential equations are derived from physical situations. The linear differential equation solution is relatively simple, but finding an analytical analysis of a nonlinear differential equation could be difficult in many situations. Therefore, because most differential equations do not have an exact closed form solution, approximation and numerical approaches are regularly employed. Temporarily, many non-linear equations do not have a small parameter, but any traditional perturbation technique requires it. Therefore, this difficulty constricts the use of these perturbation techniques. A small parameter determination is a difficult procedure that requires an implementation of special procedures. Aimed at explaining ordinary nonlinear differential equations, the semi-analytical HPM can be a useful tool. He^11^ was the first Mathematician who proposed this method to solve nonlinear differential equations. The HPM has all the benefits of the perturbation approach without any necessity for a small parametric hypothesis. This approach overcomes calculation complexity, requires less computer memory, and has a faster calculation time than the previous methods. Accordingly, it is simple, powerful, effective and promising. The method requires initial conditions and generates an indefinite numerical as an analytical approximation. The HPM has been employed to analyze nonlinear differential equations in a number of investigations. The HPM was adapted by El-Dib and Moatimid^12^ to find accurate solutions for various forms in linear and nonlinear differential equations. The primary idea in their approach is coming up with an appropriate trial function, which is commonly expressed in terms of a power series. The cancellation of the first-order approximation solution ensures that all the advanced levels are likewise ignored. Consequently, the accuracy of the fixed zero-order solution will be confirmed the exact solution. The HPM was utilized by Moatimid^13^ to get an analytical approximate solution for a sliding bead in a smooth parabola. Due to the motivation for analyzing the Duffing oscillator on a variety of physics and engineering processes, the stability of a Duffing oscillator was analyzed by Moatimid^14^. It should be noticed that the present problem differs from those revealed by Moatimid^13,14^ in the structure of the model and well as the stability analysis. Additionally, the presented perturbed solution has been verified by RK4, and this has not done previously. Using the HPM, the principal equation of motion, the stability analysis, and many analytical approximate solutions were developed. The same method is utilized by Amer et al.^15^, and He et al.^16^ to obtain the desired approximate solutions. Tian and Wang^17^ developed a stability problem of linear time-delay system. Firstly, they described a generalized vector multiple integral inequality that can interpret several results as exceptional circumstances. Secondly, a delay-dependent stability (DDS) criterion for time-delay systems was constructed using these multiples. The DDS problem for a time-varying delay linear system was proposed by Tian and Wang^18^. They demonstrated that their method is more practical for dealing with time-varying delay systems. The authors provided a numerical example to demonstrate the utility of the stability criterion.

In accordance with the above-mentioned features together with the pendulum potential applications in physics, engineering, and applied mechanics, the present study focuses on examining the motion of a pendulum coupled to a rolling wheel that is connected by a lightweight spring. To simplify the presentation, the remainder of the manuscript is systematized as follows: To help the reader, “Organization of the model” stresses on the derivation of the fundamental equation of motion. In “A bounded analytical approximate solution”, a modified analytical bounded approximate solution, based on the expanded frequency is presented. The time history of this solution is graphically represented and associated with the numerical results of the equation of motion. The comparison displays great uniformity between both solutions. The graphed phase plane show that the accomplished solutions have a stable behavior. The relation between the expanded and the natural frequency is plotted for different values of the wheel radius. In addition to the variation of the solution via the natural frequency, the linearized stability analysis is depicted throughout “Linearized stability”. The phase portraits are drawn in this Section. The findings of the whole examination are summarized as concluding remarks in “Concluding remarks”.

## Organization of the model

**Figure 1:**
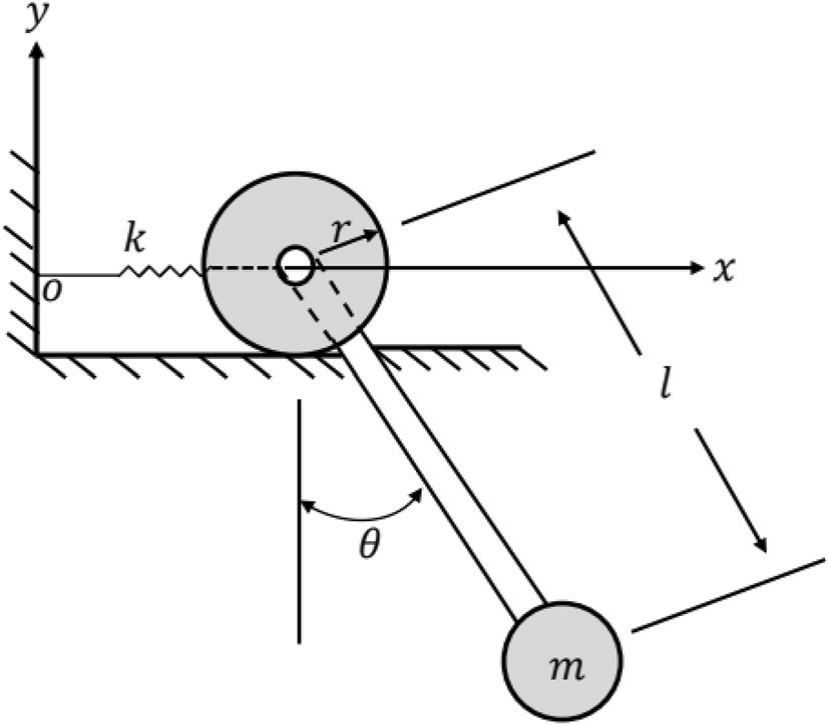
Sketches the dynamical model.

The movement of a pendulum attached to a rolling wheel that is restricted by a lightweight spring as seen in Fig. 1.

For a better fit, the Cartesian coordinates are used, where the $x$-axis is considered parallel to the horizon and *y*-axis is vertically upwards.

The *x*-axis of the given system may be inscribed as:

1 $$ x = x_{wheel} + x_{pend.} = r\theta + l\sin \theta . $$

The *y*-axis of the assumed system may be formulated as:

2 $$y = - l\cos \theta.$$

Consequently, the location of the bob is specified by

3 $$\underline{r} = \left( {r\theta + l\sin \theta , - l\cos \theta } \right)\,\,\,\,\,.$$

The kinetic energy of the system is given by

4 $$T = \frac{m}{2}\left( {r^{2} + l^{2} + 2rl\cos \theta } \right)\dot{\theta }^{2} .$$

The potential energy is given by

5 $$V = \frac{1}{2}kx_{wheel}^{2} - mgl\cos \theta .$$

The Lagrangian of the system is given by

6 $$L = \frac{m}{2}\left( {r^{2} + l^{2} + 2rl\cos \theta } \right)\dot{\theta }^{2} - \frac{1}{2}kr^{2} \theta^{2} + mgl\cos \theta .$$

Therefore, the organization takes one degree of freedom. Therefore, the equation of motion, then becomes

7 $$\frac{d}{dt}\left( {\frac{\partial L}{{\partial \dot{\theta }}}} \right) - \frac{\partial L}{{\partial \theta }} = 0,$$

Or

8 $$m\left( {r^{2} + l^{2} + 2rl\cos \theta } \right)\ddot{\theta } - mrl\dot{\theta }^{2} \sin \theta + kr^{2} \theta + mgl\sin \theta = 0.$$

Similar derivations of Eq. (8) was earlier given by Nayfeh^19^ in several situations for various mechanical problems, and by El-Dib and Moatimid^21^ for a rocking rigid rod over a circular surface.

To shorten the governing equation of motion (8), for more opportuneness, and in order to avoid scaling effects, the following non-dimensional parameters are adapted. For this purpose, consider $l$, $\sqrt {l/g}$ and $m$ as the characteristic length, time, and mass, correspondingly. It follows that Eq. (8) may be written as follows:

9 $$\left( {r^{2} + 1 + 2r\cos \theta } \right)\ddot{\theta } - r\dot{\theta }^{2} \sin \theta + \sin \theta + kr^{2} \theta = 0.$$

On expanding the Taylor expansion, for small values of $\theta$, one may consider $\sin \theta \cong \theta - \theta^{3} /3!$, and $\cos \theta \cong 1 - \theta^{2} /2!$. Equation (9) is then established as

10 $$\left( {1 - \alpha \theta^{2} } \right)\ddot{\theta } + \omega^{2} \theta - \alpha \theta \dot{\theta }^{2} + \frac{\alpha }{6}\theta^{3} \dot{\theta }^{2} - \beta \theta^{3} = 0.$$

where, $$\alpha = \frac{r}{{(r + 1)^{2} }}$$, $$\omega^{2} = \frac{{kr^{2} + 1}}{{(r + 1)^{2} }}$$, and $$\beta = \frac{1}{{6(r + 1)^{2} }}$$.

## A bounded analytical approximate solution

As previously shown, the fundamental equation of motion (10) is a nonlinear one. In reality, it has no bearing an exact closed form solution. Subsequently, it should be scrutinized by a perturbation technique. In accordance with our previous works, as stated by Moatimid^13,14^, the traditional HPM generates secular terms that are physically inconvenient. As a result, a nonlinear expanded frequency adaptation to the HPM is proposed. In this case, the Homotopy equation could be written as follows to achieve this goal

11 $$\ddot{\theta } + \omega^{2} \theta + \rho \left( { - \alpha \theta^{2} \ddot{\theta } - \alpha \theta \dot{\theta }^{2} + \frac{\alpha }{6}\theta^{3} \dot{\theta }^{2} - \beta \theta^{3} } \right) = 0,\,\,\,\,\rho \in \left[ {0,\,\,1} \right].$$

To attain a perturbed solution, for more appropriateness, one may accept that the following initial conditions: $\,\,\theta (0) = 1$ and $\dot{\theta }(0) = 0$.

By the technique of the previous comprehensive work of Moatimid^13,14^, the recognized natural frequency $\omega^{2}$ may be expanded as follows:

12 $$\Omega^{2} = \omega^{2} + \sum\limits_{i = 1}^{n} {\rho^{i} } \omega_{i} .$$

In accordance with the procedures of HPM, the time dependent function $\theta (t)$ may be expanded as follows:

13 $$\theta (t;\rho ) = \sum\limits_{i = 1}^{n} {\rho^{i} } \theta_{i} (t).$$

Allowing Laplace transforms (*L*_*T*_) of the mixtures of Eqs. (11–13), one finds

14 $$L_{T} \left\{ {\theta (t;\rho )} \right\} = \frac{s}{{s^{2} + \Omega^{2} }} - \frac{\rho }{{s^{2} + \Omega^{2} }}L_{T} \left\{ { - \alpha \theta^{2} \ddot{\theta } - \alpha \theta \dot{\theta }^{2} + \frac{\alpha }{6}\theta^{3} \dot{\theta }^{2} - \beta \theta^{3} - \left( {\omega_{1} + \rho \omega_{2} } \right)} \right\}.$$

Retaining the inverse Laplace transforms to Eq. (19), one obtains

15 $$\theta (t;\rho ) = \cos \,\Omega t - \rho L_{T}^{ - 1} \left[ {\frac{1}{{s^{2} + \Omega^{2} }}L_{T} \left\{ { - \alpha \theta^{2} \ddot{\theta } - \alpha \theta \dot{\theta }^{2} + \frac{\alpha }{6}\theta^{3} \dot{\theta }^{2} - \beta \theta^{3} - \left( {\omega_{1} + \rho \omega_{2} } \right)} \right\}} \right].$$

Subsequently, the nonlinear part may be formulated as follows:

16 $$N\left( {\sum\limits_{i}^{n} {\rho^{i} \theta_{i} } } \right) = N_{0} (\theta_{0} ) + \rho N_{1} (\theta_{0} ,\,\theta_{1} ) + \rho^{2} N_{2} (\theta_{0} ,\,\theta_{1} ,\,\theta_{2} ) + \cdots + \rho^{n} N_{n} (\theta_{0} ,\,\theta_{1} ,\,\theta_{2} ,\, \ldots ,\theta_{n} ),$$

where

17 $$N_{n} (\theta_{0} ,\,\theta_{1} ,\,\theta_{2} ,\, \ldots ,\theta_{n} ) = \frac{1}{n!}\mathop {\lim }\limits_{\rho \to 0} \frac{\partial }{{\partial \rho^{n} }}N\left( {\sum\limits_{i}^{n} {\rho^{i} \theta_{i} } } \right).$$

Employing the expansion of the time-dependent function $\theta (t;\rho )$ as is given by Eq. (13), and then equating the coefficient of like powers $\rho$ on both sides, one gets the following orders:

18 $$\rho^{0} :\theta_{0} (t) = \cos \,\Omega t,$$

19 $$\rho :\,\theta_{1} (t) = - L_{T}^{ - 1} \left[ {\frac{1}{{s^{2} + \Omega^{2} }}L_{T} \left\{ { - \alpha \theta_{0}^{2} \ddot{\theta }_{0} - \alpha \theta \dot{\theta }_{0}^{2} + \frac{\alpha }{6}\theta_{0}^{3} \dot{\theta }_{0}^{2} - \beta \theta_{0}^{3} - \omega_{1} \theta_{0} } \right\}} \right].$$

Substituting Eq. (18) into Eq. (19) to obtain a uniform expansion needs an elimination of the secular terms. Fundamentally, the coefficient of the circular function should be disappeared. Additionally, the elimination of the coefficient of the function $\cos \,\Omega t$ produces

20 $$\omega_{1} = \frac{1}{48}\left( { - 36\beta + 25\alpha \Omega^{2} } \right).$$

It follows that the uniform solution of $\theta_{1} (t)$ becoming

21 $$\theta_{1} (t) = \frac{1}{48}\left\{ { - \frac{{\left( { - 18\beta + 35\alpha \,\Omega^{2} } \right)}}{{12\Omega^{2} }}\cos \,\Omega t + \frac{{\left( { - 24\beta + 47\alpha \Omega^{2} } \right)}}{{16\Omega^{2} }}\cos \,3\Omega t - \frac{\alpha }{48}\cos \,5\Omega t} \right\}.$$

Again, the substitution of Eqs. (20) and (21) into the second order of Eq. (15), one finds that the removal of the secular term requires

22 $$\omega_{2} = \frac{1}{{18432\Omega^{2} }}\left( { - 864\beta^{2} + 1644\alpha \beta \Omega^{2} + 71\alpha^{2} \Omega^{4} - 576\beta \omega_{1} + 1120\alpha \omega_{1} \Omega^{2} } \right).$$

Therefore, the suitable solution $\theta_{2} (t)$ then becomes

23 $$\theta_{2} (t) = \frac{1}{{221184\Omega^{2} }}\left\{ \begin{gathered} - \frac{1}{{120\Omega^{2} }}\left( {103680\beta^{2} - 728280\alpha \beta \Omega^{2} + 1017157\alpha^{2} \Omega^{4} + 103680\beta \omega_{1} - 202560\alpha \omega_{1} \Omega^{2} } \right)\cos \Omega t \hfill \\ + \frac{1}{{4\Omega^{2} }}\left( {2592\beta^{2} - 17568\alpha \beta \Omega^{2} + 24307\alpha^{2} \Omega^{4} + 3456\beta \omega_{1} - 6768\alpha \omega_{1} \Omega^{2} } \right)\cos 3\Omega t \hfill \\ + \frac{1}{{12\Omega^{2} }}\left( {2592\beta^{2} - 20304\alpha \beta \Omega^{2} + 29333\alpha^{2} \Omega^{4} + 48\alpha \omega_{1} \Omega^{2} } \right)\cos 5\Omega t \hfill \\ + \frac{1}{48}\left( {720\alpha \beta - 2161\alpha^{2} \Omega^{2} } \right)\cos 7\Omega t + \frac{13}{{80}}\alpha^{2} \Omega^{2} \cos 9\Omega t \hfill \\ \end{gathered} \right\}.$$

In view of the HPM, the approximate bounded solution of the foremost equation as given in Eq. (10) may be inscribed as follows:

24 $$\theta (t) = \mathop {\lim }\limits_{\rho \to 1} \left( {\theta_{0} (t) + \rho \theta_{1} (t) + \rho^{2} \theta_{2} (t) + ...} \right),$$

where $\theta_{0} (t),\,\theta_{1} (t)$ and $\theta_{2} (t)$ are time-dependent functions given by Eqs. (18), (21) and (23), correspondingly. This approximate solution (24) necessitates that the influences of the trigonometric functions must be of a real behavior. To this end, merging Eqs. (12), (20) and (22), one attains a quadratic equation in $\Omega^{2}$ as follows:

25 $$\,\Omega^{4} + \left( {\frac{{216( - 192 + 7\alpha )\beta + 55296\omega^{2} }}{{ - 55296 + 28800\alpha + 1963\alpha^{2} }}} \right)\Omega^{2} - \frac{{1296\beta^{2} }}{{\left( { - 55296 + 28800\alpha + 1963\alpha^{2} } \right)}} = 0.$$

The required stability standards require that $\Omega^{2}$ be real and positive. The numerical calculation showed that Eq. (25) has only two real positive roots as follows: $\Omega = 0.735216$ and $\Omega = 0.009315$.

The desired stability requires that $\Omega^{2}$ is both real and positive. To confirm the updated HPM, the approximate analytical and numerical solutions are shown in a single diagram for more opportunities. As a result, for a randomly chosen system where $r = 1$ and $k = 1$, the diagram below is drawn, according to the Mathematica software (12.0.0.0), where Eq. (25) is just has a real root as $\Omega = 0.735216$.

**Figure 2:**
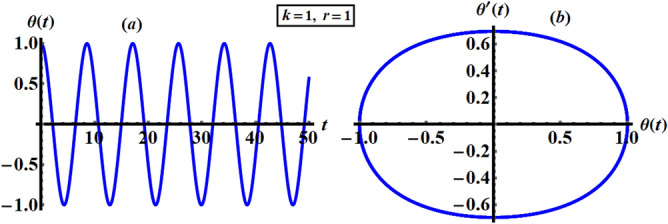
Shows the AS at $k = 1$ and $r = 1$.

The approximate solution (AS) as given in Eq. (24) has received quite a lot of attention, especially when $k = 1$ and $r = 1$. Therefore, part (*a*) in Fig. 2 shows the time history of the attained solution $\theta$ versus time $t$, meanwhile, part (b) reveals the phase plane diagram of this solution versus its first derivative $\theta^{\prime}$ at the same considered values of $k$ and $r$. A closer look at the drawn curves in this figure reveals that we have obtained a symmetric periodic wave, where its amplitude and wavelength remain stationary. The conclusion that can be made here is that the accomplished solution has a stable behavior, where the symmetric closed curve in part (*b*) asserts this statement.

**Figure 3:**
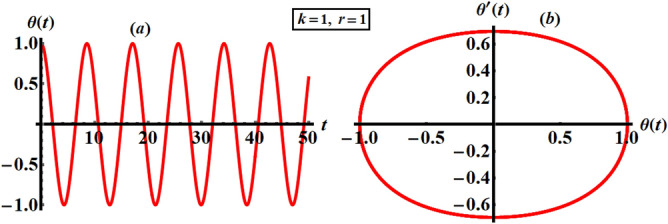
Reveals the NS at $k = 1$ and $r = 1$.

Furthermore, the numerical solution (NS) of Eq. (10) is achieved using the RK4 approach and it is graphically signified with the same mentioned values of $k$ and $r$, see Fig. 2. The displayed curves in parts (*a*) and (*b*) of this figure show the variation of $\theta$ versus time $t$ and the corresponding phase plane is graphed, respectively. It is clear that the characterized wave is periodically represented to emphasize its stable manner during the investigated period of time. This behavior is plotted versus its first derivative to yield a closed symmetric curve as in part (*b*). An inspection of the curves of Figs. 2 and 3 shows high consistency between them, which reveals the good accuracy of both solutions.

**Figure 4:**
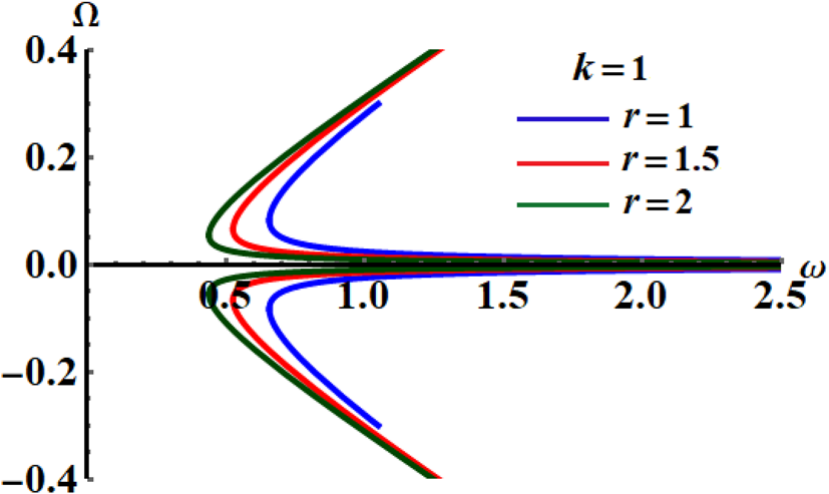
Illustrates variation of $r$ in the plane $\omega_{c} \Omega$ at $k = 1$.

The sketched curves in Fig. 4 indicate the variation of $\Omega$ via $\omega$ for various values of $r$ when $k = 2$. These trajectories are obviously symmetrical around the horizontal axis, which is in good accordance with Eq. (25).

## Linearized stability

The linearized approach is employed in this section to solve the considered autonomous system as shown in the fundamental Eq. (10). Presuming that the system is generated by the transformation $\dot{\theta } = \phi$, Eq. (10) can be divided as follows:

26 $$\begin{aligned} \dot{\theta } = f(\theta ,\,\phi ), \\ and \\  \dot{\phi } = h(\theta ,\,\phi ),\end{aligned}$$

where

27 $$ \begin{aligned} f(\theta ,\,\phi ) = \phi, \\ and \\  h(\theta ,\,\phi ) = \frac{1}{{1 - \alpha \theta^{2} }}\left( { - \omega^{2} \theta + \alpha \theta \phi^{2} - \frac{\alpha }{6}\theta^{3} \phi^{2} + \beta \theta^{3} } \right),\end{aligned}$$

28 $$\begin{aligned} f(\theta_{0} ,\,\phi_{0} ) = 0,\; \\ and \\  h(\theta_{0} ,\,\phi_{0} ) = 0. \end{aligned}$$

It follows that

29 $$\left. {\begin{array}{*{20}c} {\phi_{0} = 0} \\ { - \omega^{2} \theta + \beta \theta^{3} = 0} \\ \end{array} } \right\}.$$

**Figure 5:**
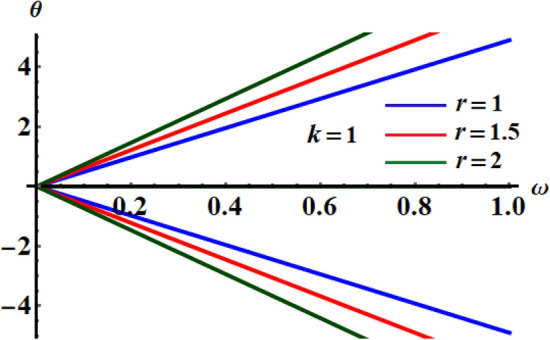
Describes the variety of $\theta$ versus $\omega$ at $r = 1,\,1.5\,\,{\text{and}}\,2$.

The drawn curves in Fig. 5 examine the variation of $\theta$ versus $\omega$ in diverse standards of $r$. These curves behave like straight lines, which is consistent with the second equation of (29). These lines start from the origin point of the plane axes $\omega \theta$ and are symmetric around the horizontal axis.

It follows that there are some of fixed points as follows:

30 $$\left. {\begin{array}{*{20}c} {\theta_{0} = 0} \\ {\theta_{0} = \pm \frac{\omega }{\sqrt \beta }} \\ \end{array} } \right\}.$$

The accompanying Jacobian array is obtained utilizing Taylor theory. The expansion of the functions $f(\theta ,\,\phi )$ and $h(\theta ,\,\phi )$ about the critical points, just keeping the linear terms, yields

31 $$J = \left( {\begin{array}{*{20}c} 0 & 1 \\ {\frac{{ - 6\beta \theta^{2} ( - 3 + \alpha \theta^{2} ) + \alpha [6 + \theta^{2} ( - 3 + \alpha (6 + \theta^{2} ))]\phi - 6(1 + \alpha \theta^{2} )\omega^{2} )}}{{6(1 - \alpha \theta^{2} )^{2} }}} & { - \frac{{\alpha \theta ( - 6 + \theta^{2} )\phi }}{{3(1 - \alpha \theta^{2} )}}} \\ \end{array} } \right).$$

On the equilibrium point, the determinant of the Jacobian matrix becomes

32 $$\left| {\begin{array}{*{20}c} { - \Lambda } & 1 \\ { - \frac{{\beta \theta^{2} ( - 3 + \alpha \theta^{2} ) + (1 + \alpha \theta^{2} )\omega^{2} }}{{(1 - \alpha \theta^{2} )^{2} }}} & { - \Lambda } \\ \end{array} } \right| = 0.$$

From the above matrix, the eigenvalues are given as:

33 $$\Lambda_{1,2} = \pm \,i\sqrt {\beta \theta^{2} ( - 3 + \alpha \theta^{2} ) + (1 + \alpha \theta^{2} )\omega^{2} } .$$

**Figure 6:**
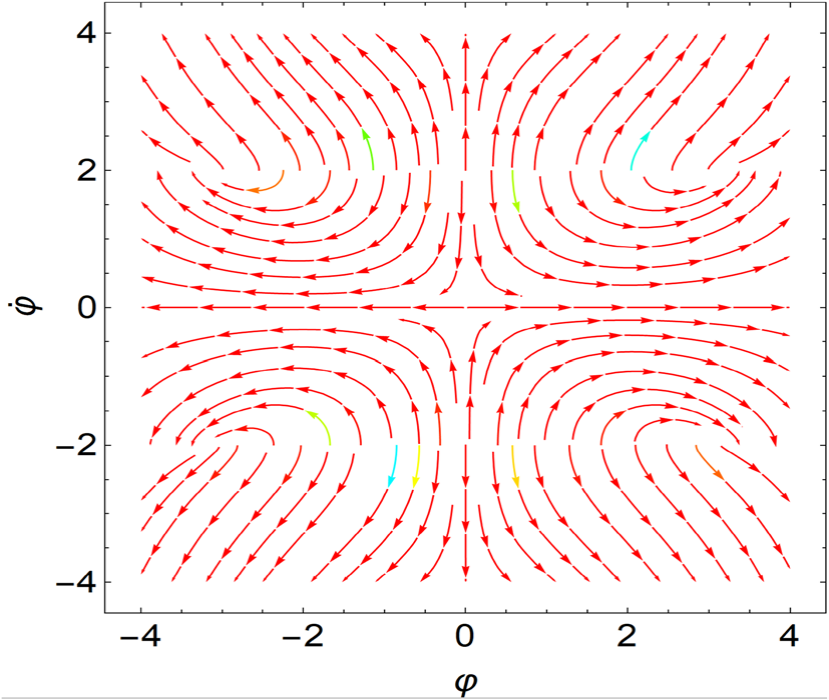
Shows a stable node at $r = 1.0,\,\,k = 3.0$.

**Figure 7:**
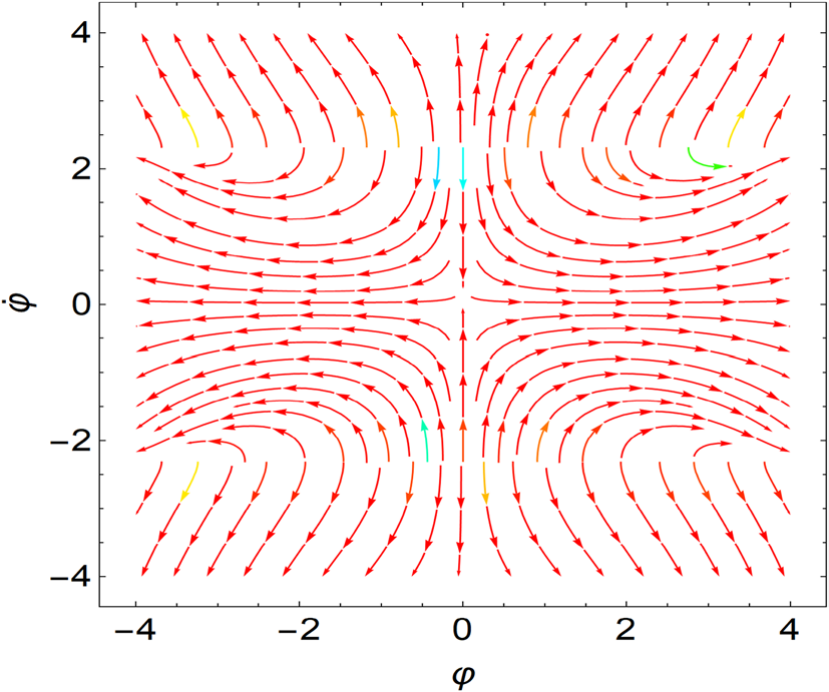
Shows a stable node at $r = 3.0,\,\,\,k = 1.0$.

If all eigenvalues of the Jacobian, which is given at the equilibrium points, require negative real parts, the equilibrium point is a stable one. Nevertheless, if at most one of the eigenvalue has a positive real part, the equilibrium point becomes unstable. It is more realistic to assume a collection of randomly particular systems to indicate the stability and instability characterizations. This procedure can be confirmed as stated in Table 1 besides the sketched curves of Figs. 6 and 7. The details are provided by Galeb et al.^21^ in our previous study. It really should be noted that the current framework in addition the stability analysis, departs from those shown by Galeb et al.^21^.

**Table 1:** Represents the equilibrium points.

	Sample chosen system	Fixed point	Roots of the Eigenvalues	Stability/unstability
1	$r = 1.0,\,\,k = 3.0$	$( \pm 4.89,\,0)$	Pure imaginary $\Lambda_{1,2} = \pm 0.63\,i$	A stable node See Fig. 6
2	$r = 3.0,\,\,\,k = 1.0$	$( \pm 7.75,\,0)$	Pure imaginary $\Lambda_{1,2} = \pm 0.35\,i$	Stable node See Fig. 7

## Concluding remarks

For so many people who are working on nonlinear differential equations, the goal is to arrive at analytical and numerical solutions. In practice, examining an approximate solution can be done in different ways. Since the basic pendulum problem is so significant in numerous zones, the current research focused on it when it was linked with a lighter spring. Therefore, the aim of this study is to investigate the movement of a pendulum on a spinning wheel, which is organized by a lightweight spring. Under certain conditions, the conserved equation of motion produced a nonlinear ordinary equation in one degree of freedom. Unfortunately, we are often unable to eliminate the presence of the sources of the secular terms that have been derived using the traditional HPM methodology. Consequently, the achieved approximate solution exhibits increasing amplitude over the time. Therefore, this method is not included in the current work. A combination of HPM and Laplace transforms is adjusted in addition to the nonlinear extending frequency to actually accomplish an approximate periodic solution. Along with the phase plane, the variation of the given solution with time is displayed. To corroborate this analytical approximate solution, numerical validations are performed. The comparison of various solutions indicates a high level of consistency, demonstrating the high precision of the used technique. The relationship between the extended and natural frequencies is clearly examined graphically. With the resources of the linearized stability investigation, the stability benchmark of the scheme is accomplished. To depict the behaviour around the fixed points, the association between the frequency of the dynamical model and solution occurs at different values of the wheel radius. Similar problems have been analyzed by Moatimid^13^, El-Dib and Moatimid^20^, and fortunately, the perturbed solution of the present case has been verified by RK4. The phase portraits are plotted for convenience to ensure the mechanism of stability and instability in the neighborhood of the equilibrium points. Generally, the current work provides the following conclusions:

- The fundamental equation of motion of the system under consideration is presented in Eq. (10).
- Eq. (24) provides an approximate periodic solution of the given problem.
- Eq. (29) is used to calculate the fixed points. Additionally, the eigenvalues are derived from Eq. (33).
- Table 1 depicts the various categories of the eigenvalues, and as a result, the behavior of stability/instability is described.

## Data Availability

Because no datasets were collected or processed during the current study, data sharing was not applicable to this paper.
